# Therapeutic Perspectives of HIV-Associated Chemokine Receptor (CCR5 and CXCR4) Antagonists in Carcinomas

**DOI:** 10.3390/ijms24010478

**Published:** 2022-12-28

**Authors:** Wilfredo Alejandro González-Arriagada, Isaac E. García, René Martínez-Flores, Sebastián Morales-Pison, Ricardo D. Coletta

**Affiliations:** 1 Facultad de Odontología, Universidad de Los Andes, Santiago 7620086, Chile; 2 Centro de Investigación e Innovación Biomédica (CIIB), Universidad de los Andes, Santiago 7620086, Chile; 3 Patología Oral y Maxilofacial, Hospital El Carmen Luis Valentín Ferrada, Maipú 9251521, Chile; 4 Laboratorio de Fisiología y Biofísica, Facultad de Odontología, Universidad de Valparaíso, Valparaíso 2360004, Chile; 5 Centro de Investigación en Ciencias Odontológicas y Médicas, Universidad de Valparaíso, Valparaíso 2360004, Chile; 6 Centro Interdisciplinario de Neurociencias de Valparaíso, Universidad de Valparaíso, Valparaíso 2381850, Chile; 7 Unidad de Patología y Medicina Oral, Facultad de Odontología, Universidad Andres Bello, Viña del Mar 2531015, Chile; 8 Centro de Oncología de Precisión (COP), Facultad de Medicina y Ciencias de la Salud, Universidad Mayor, Santiago 7560908, Chile; 9 Department of Oral Diagnosis and Graduate Program in Oral Biology, Piracicaba Dental School, University of Campinas, Piracicaba 13414-903, SP, Brazil

**Keywords:** cancer therapy, immunotherapy, chemokines, oncology

## Abstract

The interaction between malignant cells and the tumor microenvironment is critical for tumor progression, and the chemokine ligand/receptor axes play a crucial role in this process. The CXCR4/CXCL12 and CCR5/CCL5 axes, both related to HIV, have been associated with the early (epithelial–mesenchymal transition and invasion) and late events (migration and metastasis) of cancer progression. In addition, these axes can also modulate the immune response against tumors. Thus, antagonists against the receptors of these axes have been proposed in cancer therapy. Although preclinical studies have shown promising results, clinical trials are needed to include these drugs in the oncological treatment protocols. New alternatives for these antagonists, such as dual CXCR4/CCR5 antagonists or combined therapy in association with immunotherapy, need to be studied in cancer therapy.

## 1. Introduction

Cancer involves multiple events, such as uncontrolled proliferation and DNA repair failures, that trigger genetic instability, invasion, migration, angiogenesis, and metastasis. These later events depend on the interactions between malignant cells and many cells belonging to the tumor microenvironment, leading to tumor progression [1]. These processes require the activation or inhibition of different cell signaling pathways mediated by cell surface receptors and their ligands for which the chemokine ligand/receptor axes play key roles [2]. According to their chemical structure, chemokines comprise four subtypes of cytokines (C, CC, CXC, and CX3C) that act as the ligand on one or more receptors. On the other hand, chemokine receptors CR, CCR, CXCR, and CX3CR are G protein-coupled receptors activated for one or more subtypes of chemokines [3].

In cancer, and other diseases, a chemokine receptor may activate a proinflammatory or anti-inflammatory pathway, a duality exploited by neoplastic cells to improve the capacity to (i) evade the immune system, (ii) degrade the extracellular matrix, and (iii) invade the neural or vascular compartment producing metastasis [3,4]. In this sense, our great interest is the role of CXCR4/CXCL12 and CCR5/CCL5 axes in the pathogenesis of epithelial malignancies, including lung [5], gastric [6], pancreatic [7], colorectal [8], breast [9], ovarian [10], prostatic [4], hepatocellular [11], and head and neck carcinomas [12,13], as well as adenocarcinomas [14] and non-epithelial cancers such as melanoma [15], multiple myeloma [16], and lymphomas [17].

Preclinical investigations (in vitro and in vivo) have proved the efficacy of HIV-related chemokine receptor (HIVrCR) antagonists for cancer treatment. Chemokine receptor antagonist (CRA) drugs such as maraviroc (CCR5 antagonist) or plerixafor (CXCR4 antagonist) have shown a role in the suppression of cancer cell proliferation, migration, and metastasis [18,19]. Thus, the CRA-promoted blockade of these axes arises as an alternative or complementary therapy to improve outcomes in tumors resistant to radiotherapy and chemotherapy in carcinomas [11,20,21,22,23,24,25]. In this context, we aim to conduct a scoping review of the literature to describe the results of promising CRA therapies for carcinomas in order to identify the drugs to be used in the future.

## 2. Materials and Methods

This study followed the PRISMA extensions for Scoping Reviews.

### 2.1. Inclusion and Exclusion Criteria

Eligibility criteria included the articles related to CRA therapy and carcinoma published from 2010 to September 2022 in English. Clinical trials and in vitro and in vivo studies were included. Exclusion criteria were articles that do not study the role of CCR antagonists as therapy, with a focus on other drugs, with other study designs, other publication types, and with a focus on other malignancies (sarcoma or lymphoproliferative). Unpublished clinical trials were not included.

### 2.2. Search Strategy

An electronic search was performed in the following databases: PubMed/Medline, Web of Science, and Scopus. The search terms were “chemokine receptor”, “CXCR4”, “CCR5” and “cancer therapy”.

PubMed/Medline: (“chemokine”(All Fields) AND “receptor”(All Fields)) OR (“chemokine receptor”(All Fields) OR “CXCR4”(All Fields) OR “CCR5”(All Fields)) AND (“cancer”(All Fields) AND “therapy”(All Fields)) OR (“cancer therapy”(All Fields)).

Web of Science: (((ALL = (chemokine receptor)) OR ALL = (CXCR4)) OR (ALL = (CCR5) AND ALL = (cancer therapy)).

Scopus: ABS (chemokine AND receptor OR cxcr4 OR ccr5 AND cancer AND therapy).

The references of the included studies and the relevant reviews were checked for possible further studies.

### 2.3. Study Selection

The title and abstract of all the articles were identified and selected using the web tool Rayyan (https://www.rayyan.ai/, accessed on 14 October 2022). The abstracts were read independently by the authors. Controversies were resolved by discussion between the authors.

### 2.4. Data Extraction

Data were tabulated using a specially designed form in Microsoft Excel^®^. The following data were extracted: author, country, year of publication, study design (three categories: clinical trial, in vitro and in vivo studies), receptor, antagonist, adjuvant therapy, type of cancer, oncogenic mechanisms involved, effective doses in vitro, effective doses in vivo, metastasis in vivo, survival in vivo and primary tumor size in vivo.

### 2.5. Data Analysis

A qualitative synthesis of the extracted data was performed through the resume table.

## 3. Results

### 3.1. Study Selection

**Figure 1:**
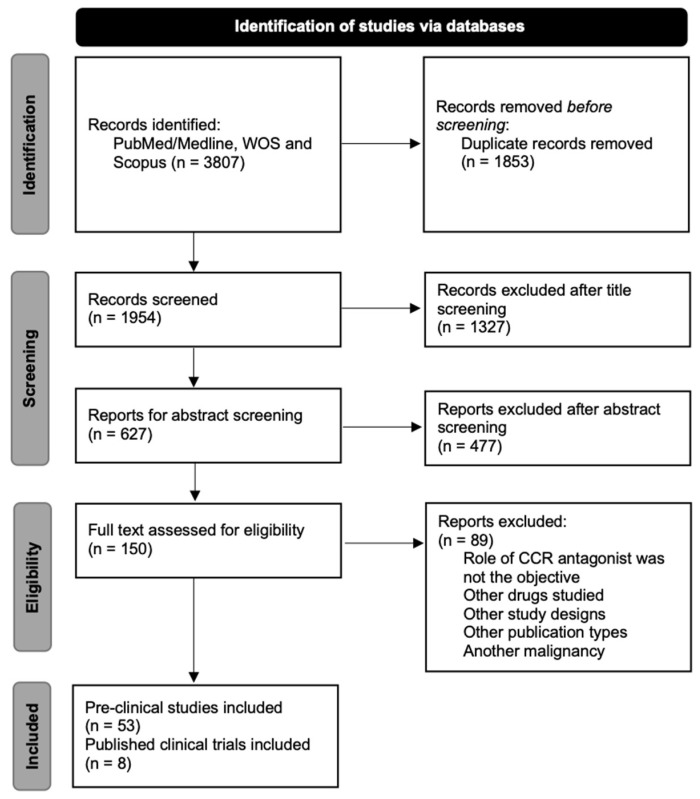
Flow diagram of the systematic selection of the studies.

A flowchart describing the selection process is presented in Figure 1. The search strategy returned 3807 articles published between 2010 and 2022 in all the databases. Duplicate records (1853 articles) were excluded, and 1954 remained for screening. After screening by title and abstract, the 150 remaining studies were assessed for eligibility with the full text. Finally, 89 articles were excluded based on the reasons detailed in Figure 1, and 61 publications were included (53 preclinical studies and 8 clinical trials).

### 3.2. Description of Preclinical Studies

Supplementary Table S1 summarizes a qualitative synthesis of the preclinical studies. Forty-three publications reported CXCR4 antagonists, and ten reported CCR5 antagonists.

We found different inhibitors reported, AMD3100 (plerixafor) and maraviroc, the most frequent for CXCR4, and CCR5, respectively. Most studies included in vitro (16 studies) and in vivo (3 studies) methodologies.

Twenty-eight studies included adjuvant therapy. Among these therapies, the most reported were doxorubicin, gemcitabine, paclitaxel, and radiation. Breast cancer was the most common epithelial cancer included. The main oncogenic mechanisms these drugs interfered with were cell invasion, migration, and metastasis. However, the modulation of the immune response is another attractive action pathway of these drugs.

### 3.3. Description of Clinical Trials

A qualitative synthesis of the published clinical trials is summarized in Supplementary Table S2. All the papers were written in English and published between 2014 and 2022. Seven clinical trials reported CXCR4 antagonists (LY2510924, BL-8040, and mavorixafor), and two clinical trials were reported with a CCR5 antagonist (maraviroc). Three studies reported their results as monotherapy, and six studies used adjuvant therapy of durvalumab, pembrolizumab, nivolumab, sunitinib, and chemotherapy (leucovorin, liposomal irinotecan, and fluorouracil). The most reported inhibitor of CXCR4 was LY2510924, with a dose of 20 mg/day reported in different protocols. The inhibitor of CCR5, maraviroc, was used at 300 mg twice daily for two months. Other reported inhibitors of CXCR4 were motixafortide and mavorixafor. All the studies reported favorable survival results.

## 4. Discussion

The present review describes the current therapeutical perspectives of HIVrCR, CCR5, and CXCR4 antagonist drugs in treating carcinomas. Chemokines are associated with leukocyte chemoattraction to lymph nodes for maturation. However, cell migration of malignant keratinocytes, leukocytes, endothelial cells, mesenchymal stem cells (MSCs), and cancer-associated fibroblasts (CAFs) arise because of the aberrant expression of chemokine receptors on the surface of cancer cells [26]. Muller et al. were the first to demonstrate that the CXCR4/CXCL12 axis has a role in tumor progression and metastasis in breast cancer [27]. The interaction between cancer cells and the factors released by the microenvironment components is crucial for epithelial cancer progression, playing a role in the early and late events of cancer progression. The migration to lymph stimulated by oncochemoattractant depends on CCR5/CCL5 and CXCR4/CXCL12 axes [12]. The implantation of metastatic islands in lymph nodes requires the preparation of a premetastatic niche, a phenomenon related to the release of CCL5 and CXCL12 by the resident cells of lymph nodes [28,29]. CCL5 and CXCL12 have been linked to cancer progression, epithelial–mesenchymal transition, immune evasion, metastasis, and worse prognosis. Therefore, using antagonists such as small molecules (e.g., maraviroc or Vicriviroc), peptide antagonists, or antibodies have been suggested as therapeutic alternatives [26,30]. The small molecules have been tested in some clinical trials for carcinomas or other inflammatory diseases and are approved for HIV patients. Even if many chemokine receptors were essential for cancer progression, available drugs against HIVrCR have shown promissory results. Additionally, because these receptors are expressed in cancer and defensive cells, these inhibitors could halt cancer progression and modulate the immune response.

### 4.1. CXCR4/CXCL12 Axis

In normal tissues, this axis is important in developmental processes, hematopoiesis, and inflammation [31] and is expressed in leukocytes, stromal fibroblasts, and endothelial cells [32]. CXC chemokine receptor 4 (CXCR4) is a G-protein-coupled receptor highly expressed in different human carcinomas [4,6,7,8,11,13,29], at all stages of the epithelial–mesenchymal transition, invasion, or metastasis [33], and has been related to a poor prognosis. Leukocytes and CAFs are sources of CXCL12 [34], a chemokine that binds to CXCR4, forming the CXCL12/CXCR4 axis. This axis has a role in different tumor pathways involved in processes such as the epithelial–mesenchymal transition, cell migration, and metastasis, including drug resistance [35,36,37].

Small molecules, such as AMD3100 (plerixafor), WZ811, LFC131, AMD070, LY2510924, X4-136, BPRCX807, and others [19,38,39,40,41,42,43,44,45,46,47,48,49,50], have been reported to inhibit CXCR4, individually or in association with other drugs or therapies (doxorubicin, cisplatin, radiation, and others) [19,40,41,48,49,50,51,52], including different carriers for a better efficacy [10,33,52,53,54,55]. Preclinical studies have shown, both in vitro and in vivo, that the inhibition of CXCR4 is effective in treating cell proliferation, angiogenesis, tumor growth, and the metastasis of different carcinoma cells [19,25,38,39,40,41,42,43,44,51,52,53,54,56,57,58,59,60,61,62,63,64,65,66,67,68,69,70,71,72,73,74,75,76], and it has been reported that CXCR4 blockade increases tumor-infiltrating lymphocytes (TILs) [77]. These drugs modify cell migration and invasion and reduce metastasis [38,40,41,54]. Additionally, they have also been demonstrated to enhance the sensitivity to chemotherapy or radiotherapy, increasing the reduction in cell viability, apoptosis, tumor growth, and metastasis, including the modulation of the crosstalk between tumor and stromal cells [19,40,41,51,52,55,78]. Finally, plerixafor can induce a better immune response against the tumor through the suppression of Treg cells and the regulation of T cell activity [37,59], and recent studies have reported that CXCR4 inhibition enhances the response to immunotherapy [50,79]. Clinical trials have provided interesting data to consider CXCR4 antagonists as an alternative in cancer therapy in multicenter, randomized, and even phase II studies. Using these drugs as coadjuvant therapy has been successful, showing acceptable safety and tolerability in patients with advanced refractory tumors and expanding the benefits of chemotherapy or immunotherapy [47,80,81,82].

### 4.2. CCR5/CCL5 Axis

The use of CCR5-inhibitor drugs in HIV patients is well tolerated, and diverse clinical outcomes have been observed as monotherapy or combined with other antiretroviral drugs (highly active antiretroviral therapy; HAART). The association of CCR5 with cancer progression is unveiling a new perspective on the use of these drugs.

CC chemokine receptor 5 (CCR5) is a G-protein-coupled receptor reported in different kinds of carcinomas, with the primary role in the late events of cancer progression, such as metastasis [13,18]. CCL3 and CCL5 are the main chemokines that bind to CCR5, forming the CCL3/CCR5 and CCL5/CCR5 activation axes. These chemokines are mainly involved in inflammation, promoting the recruitment of leukocytes to injury sites [26]. The CCR5/CCL5 axis has protumor effects, and the low expression of these proteins can lead to a better prognosis [83].

In carcinomas, some authors have described the relationship between high levels of CCL5 or CCR5 expression in tumors and advanced stages [12,13,84,85,86], including a proangiogenic role [87] and the stimulation of cancer stem cells [88]. Regarding immune modulation, CCL5 can differentiate leukocytes to a protumorigenic profile [89] and can inhibit the antitumorigenic role of CD8+ lymphocytes [90] but can also modulate the activation of Tregs [18] and myeloid-derived suppressor cells [91]. CCL5 was reported as an inducer of cell migration and invasion [92], leading to metastasis [93]. The aggressiveness of CCL5-releasing tumors relies on the fact that they are more aggressive because CCL5 promotes invasion, migration, and metastasis in CCR5-high-expressing tumors.

CCR5 inhibition has demonstrated promising results in controlling cancer development and progression in preclinical studies [18,20,22,93,94,95,96,97]. Maraviroc is a specific small-molecule antagonist of the CCR5 used in preclinical and clinical cancer studies. CCR5 inhibitors were tested to treat liver, pancreatic, and breast cancer cells, showing apoptosis induction, reduced cell invasion and metastasis, and increased survival [20,93,98]. In addition, some studies reported that CCR5 inhibition could modulate the immune response, diminishing Treg infiltration [99,100].

It was reported that the inhibition of CCR5 in colorectal cancer cells, as a single agent, can inhibit proliferation and migration but failed to inhibit metastasis in vivo [101]. However, a study reported that maraviroc could inhibit metastasis in an animal model of colorectal cancer. These contradictory results are probably related to the promiscuity of chemokine receptors and chemokines, suggesting that drugs with dual or multiple inhibitions, or combined therapies (immunotherapy or chemotherapy), could have a better effect against cancer progression and metastasis. Recent preclinical studies have reported that the combination of CCR5 antagonists with anti-PD-L1 can inhibit tumor growth and enhance the therapy outcome in several types of cancer [100,102]. A few reported clinical trials are using CCR5 antagonists [103]. They are in phase I and use maraviroc.

### 4.3. New Perspectives

**Figure 2:**
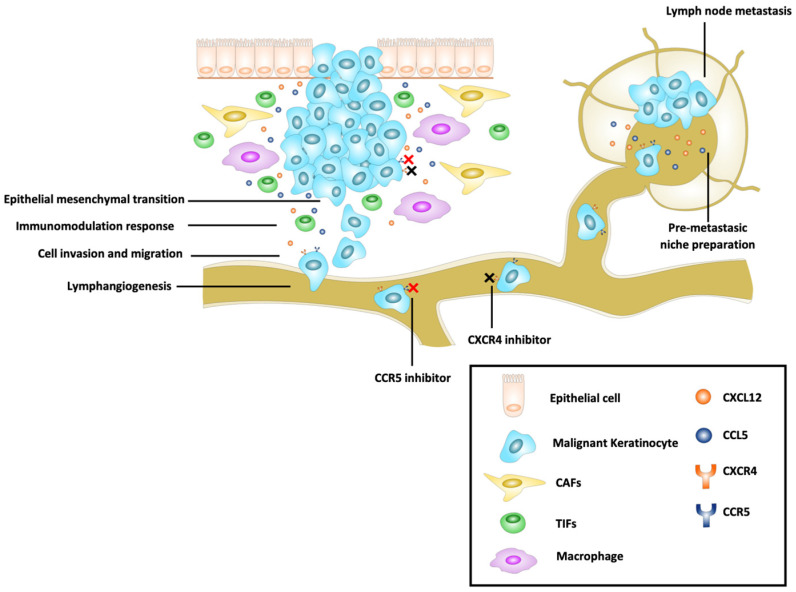
Schematic representation of oncogenic mechanisms mediated by the CCR5/CCL5 and the CXCR4/CXCL12 axes in carcinomas. The primary local sources of CCL5 and CXCL12 are CAFs, TILs, cells, macrophages, and tumor cells, while in lymph nodes, the primary sources are lymphatic fibroblasts, endothelial cells, lymphocytes, and tumor cells. These axes have a role in early events, such as epithelial–mesenchymal transition, local immune response modulation, local cell invasion, migration, and lymphangiogenesis, and in late events, such as premetastatic niche preparation, migration to lymph nodes (oncochemotaxis), and lymph node metastasis.

These chemokine axes play essential roles in cell migration and metastasis but can also modulate the immune response against tumors (Figure 2). Thus, studies about these axes as therapeutic targets are promising. The development of dual-inhibition drugs for CXCR4 and CCR5, and their association with immunotherapy, are the two main challenges in recent times. Therapies targeting CXCR4 and CCR5 have been reported as probable effective strategies for cancer. AMD3451 is the first reported dual CXCR4/CCR5 antagonist [104], but studies testing these drugs are necessary. Other drugs have also demonstrated a dual effect (e.g., NF279, penicillixanthone A, and GUT-70 [105]). The efficacy of these dual-inhibition drugs is still unknown in cancer.

A few reported clinical trials are testing these drugs in cancer, mainly in association with other therapies [45,46,47,106,107,108], and the results are still confusing. Hainsworth et al. reported a well-documented clinical trial with LY2510924 (anti-CXCR4) associated with sunitinib in metastatic renal cell carcinoma, showing promising results for this novel therapy [46]. On the other hand, clinical trials with maraviroc (antagonist of CCR5) and plerixafor (antagonist of CXCR4) have been reported, either as monotherapy or in combination with other drugs, but the literature mainly shows preclinical results in vitro or in vivo. Recent studies suggest that the chemokine receptor–ligand axis can modulate the tumor immune microenvironment [79]. Moreover, combining CXCR4 or CCR5 antagonists with anti-PD1 immunotherapy has shown promising results in colorectal cancer, renal cell carcinoma, and pancreatic ductal adenocarcinoma, including clinical trials [77,103,104,105,106,108]. These results reveal new alternatives to this therapy for human carcinomas.

## 5. Conclusions

The HIV-associated chemokine receptor (CCR5 and CXCR4) antagonists have critical roles in inhibiting cell migration, invasion, and metastasis and can modulate the tumoral immune response through Treg regulation. Preclinical studies are promising, but more clinical trials are needed. Future studies are required to test the efficacy of dual CXCR4/CCR5 antagonists and their association with immunotherapy.

## Data Availability

Not applicable.

## Supplementary Materials

The following supporting information can be downloaded at: https://www.mdpi.com/article/10.3390/ijms24010478/s1, Table S1: Description of preclinical studies; Table S2: Description of clinical trials.

## References

[1] Hanahan D. (2022). Hallmarks of Cancer: New Dimensions. Cancer Discov..

[2] Raza S., Rajak S., Tewari A., Gupta P., Chattopadhyay N., Sinha R.A., Chakravarti B. (2022). Multifaceted role of chemokines in solid tumors: From biology to therapy. Semin. Cancer Biol..

[3] Korbecki J., Grochans S., Gutowska I., Barczak K., Baranowska-Bosiacka I. (2020). CC Chemokines in a Tumor: A Review of Pro-Cancer and Anti-Cancer Properties of Receptors CCR5, CCR6, CCR7, CCR8, CCR9, and CCR10 Ligands. Int. J. Mol. Sci..

[4] Parol-Kulczyk M., Gzil A., Ligmanowska J., Grzanka D. (2022). Prognostic significance of SDF-1 chemokine and its receptors CXCR4 and CXCR7 involved in EMT of prostate cancer. Cytokine.

[5] Wang Y., Lan W., Xu M., Song J., Mao J., Li C., Du X., Jiang Y., Li E., Zhang R. (2021). Cancer-associated fibroblast-derived SDF-1 induces epithelial-mesenchymal transition of lung adenocarcinoma via CXCR4/beta-catenin/PPARdelta signalling. Cell Death Dis..

[6] Perrot-Applanat M., Vacher S., Pimpie C., Chemlali W., Derieux S., Pocard M., Bieche I. (2019). Differential gene expression in growth factors, epithelial mesenchymal transition and chemotaxis in the diffuse type compared with the intestinal type of gastric cancer. Oncol. Lett..

[7] Singh S.K., Mishra M.K., Eltoum I.A., Bae S., Lillard J.W., Singh R. (2018). CCR5/CCL5 axis interaction promotes migratory and invasiveness of pancreatic cancer cells. Sci. Rep..

[8] Ucuncu M., Serilmez M., Sari M., Bademler S., Karabulut S. (2019). The Diagnostic Significance of PDGF, EphA7, CCR5, and CCL5 Levels in Colorectal Cancer. Biomolecules.

[9] Dayer R., Babashah S., Jamshidi S., Sadeghizadeh M. (2018). Upregulation of CXC chemokine receptor 4-CXC chemokine ligand 12 axis ininvasive breast carcinoma: A potent biomarker predicting lymph node metastasis. J. Cancer Res. Ther..

[10] Xue J., Li R., Gao D., Chen F., Xie H. (2020). CXCL12/CXCR4 Axis-Targeted Dual-Functional Nano-Drug Delivery System Against Ovarian Cancer. Int. J. Nanomed..

[11] Singh S.K., Mishra M.K., Rivers B.M., Gordetsky J.B., Bae S., Singh R. (2020). Biological and Clinical Significance of the CCR5/CCL5 Axis in Hepatocellular Carcinoma. Cancers.

[12] Domingueti C.B., Janini J.B., Paranaiba L.M., Lozano-Burgos C., Olivero P., Gonzalez-Arriagada W.A. (2019). Prognostic value of immunoexpression of CCR4, CCR5, CCR7 and CXCR4 in squamous cell carcinoma of tongue and floor of the mouth. Med. Oral Patol. Oral Cir. Bucal.

[13] Gonzalez-Arriagada W.A., Lozano-Burgos C., Zuniga-Moreta R., Gonzalez-Diaz P., Coletta R.D. (2018). Clinicopathological significance of chemokine receptor (CCR1, CCR3, CCR4, CCR5, CCR7 and CXCR4) expression in head and neck squamous cell carcinomas. J. Oral Pathol. Med..

[14] Gao T., Shen Z., Ma C., Li Y., Kang X., Sun M. (2018). The CCL5/CCR5 Chemotactic Pathway Promotes Perineural Invasion in Salivary Adenoid Cystic Carcinoma. J. Oral Maxillofac. Surg..

[15] McConnell A.T., Ellis R., Pathy B., Plummer R., Lovat P.E., O’Boyle G. (2016). The prognostic significance and impact of the CXCR4-CXCR7-CXCL12 axis in primary cutaneous melanoma. Br. J. Dermatol..

[16] Beider K., Bitner H., Leiba M., Gutwein O., Koren-Michowitz M., Ostrovsky O., Abraham M., Wald H., Galun E., Peled A. (2014). Multiple myeloma cells recruit tumor-supportive macrophages through the CXCR4/CXCL12 axis and promote their polarization toward the M2 phenotype. Oncotarget.

[17] Pansy K., Feichtinger J., Ehall B., Uhl B., Sedej M., Roula D., Pursche B., Wolf A., Zoidl M., Steinbauer E. (2019). The CXCR4-CXCL12-Axis Is of Prognostic Relevance in DLBCL and Its Antagonists Exert Pro-Apoptotic Effects In Vitro. Int. J. Mol. Sci..

[18] Halvorsen E.C., Hamilton M.J., Young A., Wadsworth B.J., LePard N.E., Lee H.N., Firmino N., Collier J.L., Bennewith K.L. (2016). Maraviroc decreases CCL8-mediated migration of CCR5+ regulatory T cells and reduces metastatic tumor growth in the lungs. OncoImmunology.

[19] Chaudary N., Pintilie M., Jelveh S., Lindsay P., Hill R.P., Milosevic M. (2017). Plerixafor Improves Primary Tumor Response and Reduces Metastases in Cervical Cancer Treated with Radio-Chemotherapy. Clin. Cancer Res..

[20] Pervaiz A., Zepp M., Georges R., Bergmann F., Mahmood S., Faiza S., Berger M.R., Adwan H. (2021). Antineoplastic effects of targeting CCR5 and its therapeutic potential for colorectal cancer liver metastasis. J. Cancer Res. Clin. Oncol..

[21] Huang H., Zepp M., Georges R.B., Jarahian M., Kazemi M., Eyol E., Berger M.R. (2020). The CCR5 antagonist maraviroc causes remission of pancreatic cancer liver metastasis in nude rats based on cell cycle inhibition and apoptosis induction. Cancer Lett..

[22] Pervaiz A., Zepp M., Mahmood S., Ali D.M., Berger M.R., Adwan H. (2019). CCR5 blockage by maraviroc: A potential therapeutic option for metastatic breast cancer. Cell Oncol..

[23] Toyoma S., Suzuki S., Kawasaki Y., Yamada T. (2020). SDF-1/CXCR4 induces cell invasion through CD147 in squamous cell carcinoma of the hypopharynx. Oncol. Lett..

[24] Yoshida S., Kawai H., Eguchi T., Sukegawa S., Oo M.W., Anqi C., Takabatake K., Nakano K., Okamoto K., Nagatsuka H. (2019). Tumor Angiogenic Inhibition Triggered Necrosis (TAITN) in Oral Cancer. Cells.

[25] Taromi S., Kayser G., Catusse J., von Elverfeldt D., Reichardt W., Braun F., Weber W.A., Zeiser R., Burger M. (2016). CXCR4 antagonists suppress small cell lung cancer progression. Oncotarget.

[26] Weitzenfeld P., Ben-Baruch A. (2014). The chemokine system, and its CCR5 and CXCR4 receptors, as potential targets for personalized therapy in cancer. Cancer Lett..

[27] Muller A., Homey B., Soto H., Ge N., Catron D., Buchanan M.E., McClanahan T., Murphy E., Yuan W., Wagner S.N. (2001). Involvement of chemokine receptors in breast cancer metastasis. Nature.

[28] Chin A.R., Wang S.E. (2016). Cancer tills the Premetastatic Field: Mechanistic Basis and Clinical Implications. Clin. Cancer Res..

[29] Wang H., Pan J., Barsky L., Jacob J.C., Zheng Y., Gao C., Wang S., Zhu W., Sun H., Lu L. (2021). Characteristics of pre-metastatic niche: The landscape of molecular and cellular pathways. Mol. Biomed..

[30] Jiao X., Wang M., Zhang Z., Li Z., Ni D., Ashton A.W., Tang H.-Y., Speicher D.W., Pestell R.G. (2021). Leronlimab, a humanized monoclonal antibody to CCR5, blocks breast cancer cellular metastasis and enhances cell death induced by DNA damaging chemotherapy. Breast Cancer Res..

[31] Sharma M., Afrin F., Satija N., Tripathi R.P., Gangenahalli G.U. (2011). Stromal-Derived Factor-1/CXCR4Signaling: Indispensable Role in Homing and Engraftment of Hematopoietic Stem Cells in Bone Marrow. Stem Cells Dev..

[32] Guo F., Wang Y., Liu J., Mok S.C., Xue F., Zhang W. (2015). CXCL12/CXCR4: A symbiotic bridge linking cancer cells and their stromal neighbors in oncogenic communication networks. Oncogene.

[33] Yang X., Gao F., Zhang W., Li H., Huang X., Wei J., Bian J., Yang Y., Qian C., Sun M. (2020). “Star” miR-34a and CXCR4 antagonist based nanoplex for binary cooperative migration treatment against metastatic breast cancer. J. Control. Release.

[34] Orimo A., Gupta P.B., Sgroi D.C., Arenzana-Seisdedos F., Delaunay T., Naeem R., Carey V.J., Richardson A.L., Weinberg R.A. (2005). Stromal fibroblasts present in invasive human breast carcinomas promote tumor growth and angiogenesis through elevated SDF-1/CXCL12 secretion. Cell.

[35] Dillenburg-Pilla P., Patel V., Mikelis C.M., Zarate-Blades C.R., Doci C.L., Amornphimoltham P., Wang Z., Martin D., Leelahavanichkul K., Dorsam R.T. (2015). SDF-1/CXCL12 induces directional cell migration and spontaneous metastasis via a CXCR4/Galphai/mTORC1 axis. FASEB J..

[36] Zhang F., Cui J.Y., Gao H.F., Yu H., Gao F.F., Chen J.L., Chen L. (2020). Cancer-associated fibroblasts induce epithelial-mesenchymal transition and cisplatin resistance in ovarian cancer via CXCL12/CXCR4 axis. Future Oncol..

[37] Biasci D., Smoragiewicz M., Connell C.M., Wang Z., Gao Y., Thaventhiran J.E.D., Basu B., Magiera L., Johnson T.I., Bax L. (2020). CXCR4 inhibition in human pancreatic and colorectal cancers induces an integrated immune response. Proc. Natl. Acad. Sci. USA.

[38] Chittasupho C., Anuchapreeda S., Sarisuta N. (2017). CXCR4 targeted dendrimer for anti-cancer drug delivery and breast cancer cell migration inhibition. Eur. J. Pharm. Biopharm..

[39] Dragoj M., Bankovic J., Sereti E., Stojanov S.J., Dimas K., Pesic M., Stankovic T. (2017). Anti-invasive effects of CXCR4 and FAK inhibitors in non-small cell lung carcinomas with mutually inactivated p53 and PTEN tumor suppressors. Investig. New Drugs.

[40] Zhou K.X., Xie L.H., Peng X., Guo Q.M., Wu Q.Y., Wang W.H., Zhang G.L., Wu J.F., Zhang G.J., Du C.W. (2018). CXCR4 antagonist AMD3100 enhances the response of MDA-MB-231 triple-negative breast cancer cells to ionizing radiation. Cancer Lett..

[41] Fang X., Xie H., Duan H., Li P., Yousaf M., Xu H., Yang Y., Wang C. (2017). Anti-tumor activity of nanomicelles encapsulating CXCR4 peptide antagonist E5. PLoS ONE.

[42] He W., Yang T., Gong X.H., Qin R.Z., Zhang X.D., Liu W.D. (2018). Targeting CXC motif chemokine receptor 4 inhibits the proliferation, migration and angiogenesis of lung cancer cells. Oncol. Lett..

[43] Shen D., Zhu L., Liu Y., Peng Y., Lan M., Fang K., Guo Y. (2020). Efficacy evaluation and mechanism study on inhibition of breast cancer cell growth by multimodal targeted nanobubbles carrying AMD070 and ICG. Nanotechnology.

[44] Uchida D., Kuribayashi N., Kinouchi M., Sawatani Y., Shimura M., Mori T., Hasegawa T., Miyamoto Y., Kawamata H. (2018). Effect of a novel orally bioavailable CXCR4 inhibitor, AMD070, on the metastasis of oral cancer cells. Oncol. Rep..

[45] Galsky M.D., Vogelzang N.J., Conkling P., Raddad E., Polzer J., Roberson S., Stille J.R., Saleh M., Thornton D. (2014). A Phase I Trial of LY2510924, a CXCR4 Peptide Antagonist, in Patients with Advanced Cancer. Clin. Cancer Res..

[46] Hainsworth J.D., Reeves J.A., Mace J.R., Crane E.J., Hamid O., Stille J.R., Flynt A., Roberson S., Polzer J., Arrowsmith E.R. (2016). A Randomized, Open-Label Phase 2 Study of the CXCR4 Inhibitor LY2510924 in Combination with Sunitinib Versus Sunitinib Alone in Patients with Metastatic Renal Cell Carcinoma (RCC). Target. Oncol..

[47] O’Hara M.H., Messersmith W., Kindler H., Zhang W., Pitou C., Szpurka A.M., Wang D., Peng S.-B., Vangerow B., Khan A.A. (2020). Safety and Pharmacokinetics of CXCR4 Peptide Antagonist, LY2510924, in Combination with Durvalumab in Advanced Refractory Solid Tumors. J. Pancreat. Cancer.

[48] Zhou J., Le K., Xu M., Ming J., Yang W., Zhang Q., Lu L., Xi Z., Ruan S., Huang T. (2020). CXCR4 Antagonist AMD3100 Reverses the Resistance to Tamoxifen in Breast Cancer via Inhibiting AKT Phosphorylation. Mol. Ther.-Oncolytics.

[49] Chaudary N., Hill R.P., Stulik L., Milosevic M. (2021). The Oral CXCR4 Inhibitor X4-136 Improves Tumor Control and Reduces Toxicity in Cervical Cancer Treated with Radiation Therapy and Concurrent Chemotherapy. Int. J. Radiat. Oncol.*Biol.*Phys..

[50] Song J.S., Chang C.C., Wu C.H., Dinh T.K., Jan J.J., Huang K.W., Chou M.C., Shiue T.Y., Yeh K.C., Ke Y.Y. (2021). A highly selective and potent CXCR4 antagonist for hepatocellular carcinoma treatment. Proc. Natl. Acad. Sci. USA.

[51] Dragoj M., Milosevic Z., Bankovic J., Tanic N., Pesic M., Stankovic T. (2017). Targeting CXCR4 and FAK reverses doxorubicin resistance and suppresses invasion in non-small cell lung carcinoma. Cell Oncol..

[52] Tang S., Hang Y., Ding L., Tang W., Yu A., Zhang C., Sil D., Xie Y., Oupicky D. (2021). Intraperitoneal siRNA Nanoparticles for Augmentation of Gemcitabine Efficacy in the Treatment of Pancreatic Cancer. Mol. Pharm..

[53] Liu J.-Y., Chiang T., Liu C.-H., Chern G.-G., Lin T.-T., Gao D.-Y., Chen Y. (2015). Delivery of siRNA Using CXCR4-targeted Nanoparticles Modulates Tumor Microenvironment and Achieves a Potent Antitumor Response in Liver Cancer. Mol. Ther..

[54] Zhang F., Gong S., Wu J., Li H., Oupicky D., Sun M. (2017). CXCR4-Targeted and Redox Responsive Dextrin Nanogel for Metastatic Breast Cancer Therapy. Biomacromolecules.

[55] Fang X., Zhang K., Jiang M., Ma L., Liu J., Xu H., Yang Y., Wang C. (2021). Enhanced lymphatic delivery of nanomicelles encapsulating CXCR4-recognizing peptide and doxorubicin for the treatment of breast cancer. Int. J. Pharm..

[56] Guo H., Ge Y., Li X., Yang Y., Meng J., Liu J., Wang C., Xu H. (2017). Targeting the CXCR4/CXCL12 axis with the peptide antagonist E5 to inhibit breast tumor progression. Signal Transduct Target Ther..

[57] Li H., Chen Y., Xu N., Yu M., Tu X., Chen Z., Lin M., Xie B., Fu J., Han L. (2017). AMD3100 inhibits brain-specific metastasis in lung cancer via suppressing the SDF-1/CXCR4 axis and protecting blood-brain barrier. Am. J. Transl. Res..

[58] Reeves P.M., Abbaslou M.A., Kools F.R.W., Poznansky M.C. (2017). CXCR4 blockade with AMD3100 enhances Taxol chemotherapy to limit ovarian cancer cell growth. Anticancer Drugs.

[59] Santagata S., Napolitano M., D’Alterio C., Desicato S., Maro S.D., Marinelli L., Fragale A., Buoncervello M., Persico F., Gabriele L. (2017). Targeting CXCR4 reverts the suppressive activity of T-regulatory cells in renal cancer. Oncotarget.

[60] Izumi D., Ishimoto T., Miyake K., Sugihara H., Eto K., Sawayama H., Yasuda T., Kiyozumi Y., Kaida T., Kurashige J. (2016). CXCL12/CXCR4 activation by cancer-associated fibroblasts promotes integrin beta1 clustering and invasiveness in gastric cancer. Int. J. Cancer.

[61] Zhu W.B., Zhao Z.F., Zhou X. (2018). AMD3100 inhibits epithelial–mesenchymal transition, cell invasion, and metastasis in the liver and the lung through blocking the SDF-1α/CXCR4 signaling pathway in prostate cancer. J. Cell. Physiol..

[62] Morimoto M., Matsuo Y., Koide S., Tsuboi K., Shamoto T., Sato T., Saito K., Takahashi H., Takeyama H. (2016). Enhancement of the CXCL12/CXCR4 axis due to acquisition of gemcitabine resistance in pancreatic cancer: Effect of CXCR4 antagonists. BMC Cancer.

[63] Li Z., Chen G., Ding L., Wang Y., Zhu C., Wang K., Li J., Sun M., Oupicky D. (2019). Increased Survival by Pulmonary Treatment of Established Lung Metastases with Dual STAT3/CXCR4 Inhibition by siRNA Nanoemulsions. Mol. Ther..

[64] Wang Y., Kumar S., Rachagani S., Sajja B.R., Xie Y., Hang Y., Jain M., Li J., Boska M.D., Batra S.K. (2016). Polyplex-mediated inhibition of chemokine receptor CXCR4 and chromatin-remodeling enzyme NCOA3 impedes pancreatic cancer progression and metastasis. Biomaterials.

[65] Xie Y., Wehrkamp C.J., Li J., Wang Y., Wang Y., Mott J.L., Oupický D. (2016). Delivery of miR-200c Mimic with Poly(amido amine) CXCR4 Antagonists for Combined Inhibition of Cholangiocarcinoma Cell Invasiveness. Mol. Pharm..

[66] Mayr C., Neureiter D., Pichler M., Berr F., Wagner A., Kiesslich T., Namberger K. (2015). Cytotoxic effects of chemokine receptor 4 inhibition by AMD3100 in biliary tract cancer cells: Potential drug synergism with gemcitabine. Mol. Med. Rep..

[67] Muralidharan R., Panneerselvam J., Chen A., Zhao Y.D., Munshi A., Ramesh R. (2015). HuR-targeted nanotherapy in combination with AMD3100 suppresses CXCR4 expression, cell growth, migration and invasion in lung cancer. Cancer Gene Ther..

[68] Xiang J., Hurchla M.A., Fontana F., Su X., Amend S.R., Esser A.K., Douglas G.J., Mudalagiriyappa C., Luker K.E., Pluard T. (2015). CXCR4 Protein Epitope Mimetic Antagonist POL5551 Disrupts Metastasis and Enhances Chemotherapy Effect in Triple-Negative Breast Cancer. Mol. Cancer Ther..

[69] Huang M.B., Giesler K.E., Katzman B.M., Prosser A.R., Truax V., Liotta D.C., Wilson L.J., Bond V.C. (2018). Small molecule CXCR4 antagonists block the HIV-1 Nef/CXCR4 axis and selectively initiate the apoptotic program in breast cancer cells. Oncotarget.

[70] Mei L., Liu Y., Zhang Q., Gao H., Zhang Z., He Q. (2014). Enhanced antitumor and anti-metastasis efficiency via combined treatment with CXCR4 antagonist and liposomal doxorubicin. J. Control. Release.

[71] Wong D., Kandagatla P., Korz W., Chinni S.R. (2014). Targeting CXCR4 with CTCE-9908 inhibits prostate tumor metastasis. BMC Urol..

[72] Yang Q., Zhang F., Ding Y., Huang J., Chen S., Wu Q., Wang Z., Wang Z., Chen C. (2014). Antitumour activity of the recombination polypeptide GST-NT21MP is mediated by inhibition of CXCR4 pathway in breast cancer. Br. J. Cancer.

[73] Jeong W.-J., Choi I.J., Park M.-W., An S.-Y., Jeon E.-H., Paik J.H., Sung M.-W., Ahn S.-H. (2014). CXCR4 antagonist inhibits perineural invasion of adenoid cystic carcinoma. J. Clin. Pathol..

[74] Heckmann D., Maier P., Laufs S., Wenz F., Zeller W.J., Fruehauf S., Allgayer H. (2013). CXCR4 Expression and Treatment with SDF-1alpha or Plerixafor Modulate Proliferation and Chemosensitivity of Colon Cancer Cells. Transl. Oncol..

[75] Greco S.J., Patel S.A., Bryan M., Pliner L.F., Banerjee D., Rameshwar P. (2011). AMD3100-mediated production of interleukin-1 from mesenchymal stem cells is key to chemosensitivity of breast cancer cells. Am. J. Cancer Res..

[76] Portella L., Vitale R., De Luca S., D’Alterio C., Ierano C., Napolitano M., Riccio A., Polimeno M.N., Monfregola L., Barbieri A. (2013). Preclinical development of a novel class of CXCR4 antagonist impairing solid tumors growth and metastases. PLoS ONE.

[77] Gong R., Ren H. (2020). Targeting chemokines/chemokine receptors: A promising strategy for enhancing the immunotherapy of pancreatic ductal adenocarcinoma. Signal Transduct. Target. Ther..

[78] Khan M.A., Srivastava S.K., Zubair H., Patel G.K., Arora S., Khushman M., Carter J.E., Gorman G.S., Singh S., Singh A.P. (2020). Co-targeting of CXCR4 and hedgehog pathways disrupts tumor-stromal crosstalk and improves chemotherapeutic efficacy in pancreatic cancer. J. Biol. Chem..

[79] Li Z., Wang Y., Shen Y., Qian C., Oupicky D., Sun M. (2020). Targeting pulmonary tumor microenvironment with CXCR4-inhibiting nanocomplex to enhance anti-PD-L1 immunotherapy. Sci. Adv..

[80] Bockorny B., Semenisty V., Macarulla T., Borazanci E., Wolpin B.M., Stemmer S.M., Golan T., Geva R., Borad M.J., Pedersen K.S. (2020). BL-8040, a CXCR4 antagonist, in combination with pembrolizumab and chemotherapy for pancreatic cancer: The COMBAT trial. Nat. Med..

[81] Bockorny B., Macarulla T., Semenisty V., Borazanci E., Feliu J., Ponz-Sarvise M., Abad D.G., Oberstein P., Alistar A., Muñoz A. (2021). Motixafortide and Pembrolizumab Combined to Nanoliposomal Irinotecan, Fluorouracil, and Folinic Acid in Metastatic Pancreatic Cancer: The COMBAT/KEYNOTE-202 Trial. Clin. Cancer Res..

[82] Choueiri T.K., Atkins M.B., Rose T.L., Alter R.S., Ju Y., Niland K., Wang Y., Arbeit R., Parasuraman S., Gan L. (2021). A phase 1b trial of the CXCR4 inhibitor mavorixafor and nivolumab in advanced renal cell carcinoma patients with no prior response to nivolumab monotherapy. Investig. New Drugs.

[83] Aldinucci D., Casagrande N. (2018). Inhibition of the CCL5/CCR5 Axis against the Progression of Gastric Cancer. Int. J. Mol. Sci..

[84] Ma G., Huang H., Li M., Li L., Kong P., Zhu Y., Xia T., Wang S. (2018). Plasma CCL5 promotes EMT-medicated epirubicin-resistance in locally advanced breast cancer. Cancer Biomark.

[85] Wang T., Wei Y., Tian L., Song H., Ma Y., Yao Q., Feng M., Wang Y., Gao M., Xue Y. (2016). C-C motif chemokine ligand 5 (CCL5) levels in gastric cancer patient sera predict occult peritoneal metastasis and a poorer prognosis. Int. J. Surg..

[86] Yaal-Hahoshen N., Shina S., Leider-Trejo L., Barnea I., Shabtai E.L., Azenshtein E., Greenberg I., Keydar I., Ben-Baruch A. (2006). The chemokine CCL5 as a potential prognostic factor predicting disease progression in stage II breast cancer patients. Clin. Cancer Res..

[87] Suffee N., Hlawaty H., Meddahi-Pelle A., Maillard L., Louedec L., Haddad O., Martin L., Laguillier C., Richard B., Oudar O. (2012). RANTES/CCL5-induced pro-angiogenic effects depend on CCR1, CCR5 and glycosaminoglycans. Angiogenesis.

[88] Zhang Y., Yao F., Yao X., Yi C., Tan C., Wei L., Sun S. (2009). Role of CCL5 in invasion, proliferation and proportion of CD44+/CD24- phenotype of MCF-7 cells and correlation of CCL5 and CCR5 expression with breast cancer progression. Oncol. Rep..

[89] Laubli H., Spanaus K.S., Borsig L. (2009). Selectin-mediated activation of endothelial cells induces expression of CCL5 and promotes metastasis through recruitment of monocytes. Blood.

[90] Chang L.-Y., Lin Y.-C., Mahalingam J., Huang C.-T., Chen T.-W., Kang C.-W., Peng H.-M., Chu Y.-Y., Chiang J.-M., Dutta A. (2012). Tumor-Derived Chemokine CCL5 Enhances TGF-β–Mediated Killing of CD8+ T Cells in Colon Cancer by T-Regulatory Cells. Cancer Res..

[91] Zhang Y., Lv D., Kim H.J., Kurt R.A., Bu W., Li Y., Ma X. (2013). A novel role of hematopoietic CCL5 in promoting triple-negative mammary tumor progression by regulating generation of myeloid-derived suppressor cells. Cell Res..

[92] Long H., Xie R., Xiang T., Zhao Z., Lin S., Liang Z., Chen Z., Zhu B. (2012). Autocrine CCL5 signaling promotes invasion and migration of CD133+ ovarian cancer stem-like cells via NF-kappaB-mediated MMP-9 upregulation. Stem Cells.

[93] Velasco-Velazquez M., Jiao X., De La Fuente M., Pestell T.G., Ertel A., Lisanti M.P., Pestell R.G. (2012). CCR5 antagonist blocks metastasis of basal breast cancer cells. Cancer Res..

[94] Pervaiz A., Ansari S., Berger M.R., Adwan H. (2015). CCR5 blockage by maraviroc induces cytotoxic and apoptotic effects in colorectal cancer cells. Med. Oncol..

[95] Mencarelli A., Graziosi L., Renga B., Cipriani S., D’Amore C., Francisci D., Bruno A., Baldelli F., Donini A., Fiorucci S. (2013). CCR5 Antagonism by Maraviroc Reduces the Potential for Gastric Cancer Cell Dissemination. Transl. Oncol..

[96] Sicoli D., Jiao X., Ju X., Velasco-Velazquez M., Ertel A., Addya S., Li Z., Ando S., Fatatis A., Paudyal B. (2014). CCR5 receptor antagonists block metastasis to bone of v-Src oncogene-transformed metastatic prostate cancer cell lines. Cancer Res..

[97] Ward S.T., Li K.K., Hepburn E., Weston C.J., Curbishley S.M., Reynolds G.M., Hejmadi R.K., Bicknell R., Eksteen B., Ismail T. (2014). The effects of CCR5 inhibition on regulatory T-cell recruitment to colorectal cancer. Br. J. Cancer.

[98] Ochoa-Callejero L., Perez-Martinez L., Rubio-Mediavilla S., Oteo J.A., Martinez A., Blanco J.R. (2013). Maraviroc, a CCR5 antagonist, prevents development of hepatocellular carcinoma in a mouse model. PLoS ONE.

[99] Tan M.C., Goedegebuure P.S., Belt B.A., Flaherty B., Sankpal N., Gillanders W.E., Eberlein T.J., Hsieh C.S., Linehan D.C. (2009). Disruption of CCR5-dependent homing of regulatory T cells inhibits tumor growth in a murine model of pancreatic cancer. J. Immunol..

[100] Wang J., Saung M.T., Li K., Fu J., Fujiwara K., Niu N., Muth S., Wang J., Xu Y., Rozich N. (2022). CCR2/CCR5 inhibitor permits the radiation-induced effector T cell infiltration in pancreatic adenocarcinoma. J. Exp. Med..

[101] Cambien B., Richard-Fiardo P., Karimdjee B.F., Martini V., Ferrua B., Pitard B., Schmid-Antomarchi H., Schmid-Alliana A. (2011). CCL5 neutralization restricts cancer growth and potentiates the targeting of PDGFRbeta in colorectal carcinoma. PLoS ONE.

[102] Liu C., Yao Z., Wang J., Zhang W., Yang Y., Zhang Y., Qu X., Zhu Y., Zou J., Peng S. (2019). Macrophage-derived CCL5 facilitates immune escape of colorectal cancer cells via the p65/STAT3-CSN5-PD-L1 pathway. Cell Death Differ..

[103] Haag G.M., Springfeld C., Grün B., Apostolidis L., Zschäbitz S., Dietrich M., Berger A.-K., Weber T.F., Zoernig I., Schaaf M. (2022). Pembrolizumab and maraviroc in refractory mismatch repair proficient/microsatellite-stable metastatic colorectal cancer—The PICCASSO phase I trial. Eur. J. Cancer.

[104] Princen K., Hatse S., Vermeire K., Aquaro S., De Clercq E., Gerlach L.O., Rosenkilde M., Schwartz T.W., Skerlj R., Bridger G. (2004). Inhibition of human immunodeficiency virus replication by a dual CCR5/CXCR4 antagonist. J. Virol..

[105] Grande F., Occhiuzzi M.A., Rizzuti B., Ioele G., De Luca M., Tucci P., Svicher V., Aquaro S., Garofalo A. (2019). CCR5/CXCR4 Dual Antagonism for the Improvement of HIV Infection Therapy. Molecules.

[106] Halama N., Zoernig I., Berthel A., Kahlert C., Klupp F., Suarez-Carmona M., Suetterlin T., Brand K., Krauss J., Lasitschka F. (2016). Tumoral Immune Cell Exploitation in Colorectal Cancer Metastases Can Be Targeted Effectively by Anti-CCR5 Therapy in Cancer Patients. Cancer Cell.

[107] Seo Y.D., Jiang X., Sullivan K.M., Jalikis F.G., Smythe K.S., Abbasi A., Vignali M., Park J.O., Daniel S.K., Pollack S.M. (2019). Mobilization of CD8+ T Cells via CXCR4 Blockade Facilitates PD-1 Checkpoint Therapy in Human Pancreatic Cancer. Clin. Cancer Res..

[108] Haag G.M., Halama N., Springfeld C., Grün B., Apostolidis L., Zschaebitz S., Dietrich M., Berger A.-K., Weber T.F., Zoernig I. (2020). Combined PD-1 inhibition (Pembrolizumab) and CCR5 inhibition (Maraviroc) for the treatment of refractory microsatellite stable (MSS) metastatic colorectal cancer (mCRC): First results of the PICCASSO phase I trial. J. Clin. Oncol..

