# Supplementary material for: Therapeutic Perspectives of HIV-Associated Chemokine Receptor (CCR5 and CXCR4) Antagonists in Carcinomas
Source: Int J Mol Sci. 2022 Dec 28;24(1):478. doi: 10.3390/ijms24010478 (PMC9820365; doi:10.3390/ijms24010478)
Supplement: Supplementary file 1 [file ijms-24-00478-s001.zip › ijms-2051037 supplementary.pdf]

Table S1. Description of pre-clinical studies.

Author (year)	Type of study	Receptor	Inhibitor	Coadjuvant	Type of cancer	Oncogenic mechanism involved	Effective dosis in vitro	Effective dosis in vivo	Survival in vivo	Metastasis in vivo	Primary tumor size in vivo
Greco et al. (2011)[75]	In vitro & In vivo	CXCR4	AMD3100	Carboplatino	Breast	Cell cycle arrest.	10 and 100 ng/ml	50 mg/kg	No data	No data	Reduction
Portella et al. (2013)[76]	In vitro & In vivo	CXCR4	AMD3100<br>Peptide R<br>Peptide I<br>Peptide S	None	Solid tumors	Tumor growth and metastasis.	10 μM<br>10 μM<br>10 μM<br>10 μM	1.25 mg/kg<br>2 mg/kg<br>2 mg/kg<br>2 mg/kg	No data	Reduction	Reduction
Heckmann et al. (2013)[74]	In vitro	CXCR4	AMD3100	None	Colon	Cell proliferation and metastasis.	100 μM	-	-	-	-
Mei et al. (2014)[70]	In vitro & In vivo	CXCR4	Peptido S	DOX-Lip	Metastatic	Cell invasion and metastasis.	10 μM	2 mg/kg	No data	Reduction in association with DOX	Reduction in association with DOX
Wong et al. (2014)[71]	In vitro & In vivo	CXCR4	CTCE-9908	None	Prostate	Cell growth, invasion, and metastasis.	50 μg/ml	25 mg/kg	No data	Reduction	No effect
Yang et al. (2014)[72]	In vitro & In vivo	CXCR4	GST-NT21MP	None	Breast	Cell migration and invasion, tumor growth and metastasis.	2 μg/ml	5000 μg/kg	No data	Reduction	Reduction
Jeong et al. (2014)[73]	In vitro & In vivo	CXCR4	AMD3100	None	Adenoid cystic carcinoma (Salivary gland)	Perineural invasion and lymphovascular invasion.	1000 ng/mL	5 mg/kg	No data	No data	Reduction
Liu et al. (2015)[53]	In vitro & In vivo	CXCR4	AMD3100<br>CXCR4-targeted lipid-based nanoparticle (NP)	VEGF siRNA<br>Sorafenib	Liver	Angiogenesis. Enhances sensitivity to chemotherapy.	200 μmol/l	1.5 mg/kg	No data	Reduction	Reduction
Mayr et al. (2015)[66]	In vitro	CXCR4	AMD3100	Gemcitabine	Biliary tract	Cell adhesion, invasion, metastasis, migration, and proliferation.	400 μg/ml	-	-	-	-
Muralidharan et al. (2015)[67]	In vitro	CXCR4	AMD3100	HuR-FNP	Lung	Cell cycle arrest, migration, and invasion.	100 ng/ml	-	-	-	-

Xiang et al. (2015)[68]	In vitro & In vivo	CXCR4	POL5551	Eribulin	Breast	Metastasis and tumor growth.	8 μM	20 mg/kg	No data	Reduction Improves in association with eribulin.	No reduction Improves in association with eribulin.
Izumi et al. (2016)[60]	In vitro	CXCR4	AMD3100	None	Gastric	Invasion and metastasis.	10 mg/ml	-	-	-	-
Morimoto et al. (2016)[62]	In vitro & In vivo	CXCR4	AMD11070 KRH3955	None	Pancreas	Tumor growth, invasion, and metastasis	1 μM	1 mg/kg	No data	No data	Reduction
Taromi et al. (2016)[25]	In vitro & In vivo	CXCR4	AMD3100 TN14003	Etoposide Cisplatin	Lung	Tumor growth and Metastasis.	AMD3100: 100 nM TNI4003: 5 μM	2.5 mg/kg	No data	Reduction as monotherapy and in association with CT.	Reduction as monotherapy and improved in association with CT.
Wang et al. (2016)[64]	In vitro & In vivo	CXCR4	Polymeric CXCR4 antagonist (PCX)	siNCOA3 in PCX/siNCOA3 polyplexes.	Pancreas	Tumor growth and metastasis.	1 μg/mL	Not specified	No data	Reduction	Reduction
Xie et al. (2016)[65]	In vitro	CXCR4	Polymeric CXCR4 antagonist (PCX)	miR-200c in r PCX/microRNA polyplexes.	Liver	Invasion and metastasis.	200 nM	-	-	-	-
Chittasupho et al. (2017)[38]	In vitro	CXCR4	LFC131	Doxorubicin (DOX)	Breast	Cell migration.	0.5 and 1 mg/mL	-	-	-	-
Dragoj et al. (2017a)[39]	In vitro & In vivo	CXCR4	WZ811	None	Lung	Cell migration and invasion.	1 μM for 24 h	Not specified	Median survival of 4 days longer.	No data	No data
Dragoj et al. (2017b)[51]	In vitro	CXCR4	WZ811	Doxorubicin (DOX)	Lung	Cell viability. Associated to DOX, induce cell death, and decrease gelatin degradation.	10 μM	-	-	-	-
Fang et al. (2017)[55]	In vitro	CXCR4	Peptide E5	Doxorubicin (DOX)	Breast, Liver, Pancreas, Prostate, and Cervical tumor	Cell invasion and migration. Enhances sensitivity to chemotherapy.	10 μM	-	-	-	-
Guo et al. (2017)[56]	In vitro & In vivo	CXCR4	Peptide E5	Paclitaxel Ciclofosfamida	Breast	Cell migration. Enhances sensitivity to chemotherapy.	50, 75 y 100 μM	40 mg/kg	Most significant prolonged survival	No data	Similar reduction in association with E5+CTX and E5+PTX.

									with E5+CTX.		
Li et al. (2017)[57]	In vitro & In vivo	CXCR4	AMD3100	None	Lung	Cell proliferation, migration, and invasion.	0.1 and 1 ug/ml	5 μg/kg	Median survival of 23 days longer.	Reduction	No data
Reeves et al. (2017)[58]	In vitro	CXCR4	AMD3100	Paclitaxel	Ovary	Cell proliferation.	10 μmol/l in combination with Paclitaxel (0.005 μmol/l).	-	-	-	-
Santagata et al. (2017)[59]	In vitro	CXCR4	AMD3100	None	Renal	Suppression of Tregs cells.	10 μM	-	-	-	-
Zhang et al. (2017)[54]	In vitro & In vivo	CXCR4	AMD3100	Doxorubicin (DOX) in DOX-loaded AMD3100-coated dextrin nanogel (DOX-AMD-DNG)	Breast	Cell invasion and metastasis.	0,2 μg/ml<br><br>2 mg/ml (in DOX-AMD-DNG)	2 mg/kg	No data	Significant reduction	Significant reduction
Chaudary et al. (2017)[19]	In vivo	CXCR4	AMD3100	Radiation Cisplatino	Cervical	Tumor growth and lymph node metastases	-	5 mg/kg	No data	Reduction	Reduction
Zhou et al. (2018)[40]	In vitro & In vivo	CXCR4	AMD3100	Radiation (RT)	Breast	Enhances sensitivity to ionizing radiation, inducing apoptosis.	1 μg/ml	4 mg/kg	Not specified	No data	Significant reduction in association with RT.
He et al. (2018)[42]	In vitro & In vivo	CXCR4	AMD3100	None	Lung	Cell proliferation, migration, and angiogenesis.	2 μmol/l	1.25 mg/kg	No data	Reduction	Reduction
Zhu et al. (2018)[61]	In vitro & In vivo	CXCR4	AMD3100	SDF-1α	Liver and prostate	Metastasis.	1 mg/L	No data	No data	Reduction	Reduction
Huang et al. (2018)[69]	In vitro	CXCR4	AMD3100<br>IT1T<br>MSX-122<br>TIQ-15	None	Breast	Metastasis and apoptosis	0.47 μM<br>0.0007 μM<br>12 μM<br>0.0007 μM	-	-	-	-
Uchida et al. (2018)[44]	In vitro & In vivo	CXCR4	AMD070	None	Oral cavity	Cell growth, migration, and invasion	6.6 μM	2 mg/kg	No data	Reduction	No data
Li et al. (2019)[63]	In vitro & In vivo	CXCR4	FM (AMD3100)	siSTAT3 FM@PFC/siRNA nanoemulsion.	Breast	Proliferation, angiogenesis, and metastasis.	2.5 mg/mL	1.2 mg/kg	Median survival of 5 days longer.	Reduction	No data

Biasci et al (2020)[37]	In vitro	CXCR4	AMD3100	None	Colorectal and pancreas	Immune cell and cancer cell migration.	100 ng/mL	-	-	-	-
Khan et al. (2020)[78]	In vitro & In vivo	CXCR4	AMD3100	Gemcitabine GDC-0449	Pancreas	Enhances sensitivity to chemotherapy.	AMD3100 (5 μg/ml) GDC-0449 (5 μM) Gemcitabine (5 μM)	No data	No data	No data	As monotherapy less reduction than GDC-0449, but better in association with Gemcitabine.
Li et al. (2020)[79]	In vitro & In vivo	CXCR4	FX/siPD-L1@HP	None	Lung	Modulation of immune response (dendritic cells and T cells) with elimination of immune suppression.	5 mM	0.8 mg/kg	Median survival of 30 days longer.	No data	Reduction
Shen et al. (2020)[43]	In vitro	CXCR4	AMD070	None	Breast	Cell growth and apoptosis.	12 mg/ml	-	-	-	-
Yang et al. (2020)[33]	In vitro & In vivo	CXCR4	CM	miR34a (DPC/miR-34a)	Breast	Apoptosis and metastasis.	20 μg/mL DPC/ miRNA solution. CM concentration 300 nM.	5 mg/kg DPC<br>0.5 mg/kg miR-34a	No data	No data	Reduction in DPC/miR-34a group.
Xue et al. (2020)[10]	In vitro & In vivo	CXCR4	AMD3100	Paclitaxel (AMD-NP-PTX)	Ovary	Tumor growth and metastasis.	20 mg, 0.025 mmol in AMD-NP-PTX	200 μL of NP and AMD-NP (IV)	No data	No data	Reduction
Zhou et al. (2020)[48]	In vitro	CXCR4	AMD3100	None	Breast	Enhances sensitivity to tamoxifen.	10-20-40 μM	-	-	-	-
Chaudary et al. (2021)[49]	In vivo	CXCR4	X4-136	Cisplatin	Cervix	Tumor growth and metastasis.	-	X4-136 (100 mg/kg/d orally) cisplatin (4 mg/kg/wk IP)	No data	Reduction	Reduction
Fang et al. (2021)[55]	In vitro & In vivo	CXCR4	Peptide E5	Doxurubicin (M-E5-Dox)	Breast	Enhances sensitivity to chemotherapy. Tumor growth and metastasis.	2 mL of M-E5-Dox (1 mg/mL)	E5: 30 mg/kg	No data	No data	Reduction

Song et al. (2021)[50]	In vitro & In vivo	CXCR4	BPRCX807	Sorafenib Anti-PD1	Liver	Cell migration and metastasis.	1 μM	15 mg/kg (SC)	Significantly extended in combined therapy with Sorafenib.	Reduction	Reduction
Tang et al. (2021)[52]	In vitro & In vivo	CXCR4	PAMD-CHOL	In combination with siPLK. Gemcitabine	Pancreas	Cell migration and enhances sensitivity to gemcitabine.	2 μg/mL	2.5 mg/kg	No data	Reduction	Reduction
Velasco-Velazquez et al. (2012)[93]	In vitro & In vivo	CCR5	Maraviroc Vicriviroc	None	Breast	Invasion and metastasis.	100 nmol/L	8 mg/kg	No data	Reduction	Reduction
Mencarelli et al. (2013)[95]	In vitro & In vivo	CCR5	Maraviroc	None	Gastric	Cell migration	5 μM	50 mg/kg	Increased survival	Reduction	Reduction
Sicoli et al. (2014)[96]	In vivo	CCR5	Maraviroc	None	Prostate	Cell proliferation, invasion, and metastasis.	No data	8 mg/kg	No data	Reduction	No data
Ward et al. (2014)[97]	In vitro & In vivo	CCR5	Maraviroc	None	Colorectal	Antitumoral immunity (recruitment of Tregs).	1 μM	No data	No data	No data	No data
Pervaiz et al. (2015)[94]	In vitro	CCR5	Maraviroc	None	Colorectal	Tumor growth and metastasis.	100–750 μM	_	-	-	-
Halvorsen et al. (2016)[18]	In vitro & In vivo	CCR5	Maraviroc	None	Lung	Treg cells migration and metastasis.	1 μM	31 mg/kg	No data	Reduction	No data
Pervaiz et al. (2019)[22]	In vitro & In vivo	CCR5	Maraviroc	None	Breast	Cell proliferation, migration, and metastasis.	100 μM	25 mg/kg	No data	Reduction	Reduction
Huang et al. (2020)[21]	In vitro & In vivo	CCR5	Maraviroc	None	Pancreas	Cell proliferation, downregulation of cell cycle, apoptosis, and metastasis.	50 mg/ml	15 mg/kg	No data	Reduction	No data
Pervaiz et al. (2021)[20]	In vitro & In vivo	CCR5	Maraviroc	Gemcitabine	Colorectal	Cell proliferation, migration, and metastasis.	1.5–750 μM	25 mg/kg	No data	Reduction	Reduction
Wang et al. (2022)[100]	In vitro	CCR5	CCR2/5i	Anti-PD-1	Pancreas	Cell proliferation and T-cell infiltration.	No data	20 or 50 mg/kg	Increasing	Reduction	Reduction

Table S2: Description of clinical trials.

Author (year)	Study design, patients and type of carcinoma	Receptor	Inhibitor and dose administration	Combination and dose administration	Principal study group results	Comparative or additional group results	Study conclusions

Galsky et al. (2014)[45]	- Multicenter, nonrandomized, open-label, Phase Ia, dose escalation trial<br>- Part A= 25 patients<br>- Part B= 20 patients<br>- Carcinomas of the colon, lung, breast, pancreas, and prostate.	CXCR4	- Part A (dose escalation): LY2510924 (LY) daily SCI on 28-day cycles at 1.0, 2.5, 5.0, 10, 20 and 30mg/day.<br>- Part B (dose confirmation):2.5 or 20mg/day<br>- DLT=30mg/day<br>- MTD=20mg/day<br>- Min. cycles= 1<br>- Max. cycles =12	Not required	- SD= 21 (47%)<br>- PD= 29 (64%)<br>- PR or CR= 0 (0%)<br>- Not assessed= 7 (16%)	Not required	In summary, LY2510924 interacted with the desired CXCR4 target and produced a strong pharmacodynamic response. The recommended phase II dose was 20 mg/day.
Hainsworth et al. (2016)[46]	- Multicenter, randomized, open-label, Phase II trial<br>- Arm A= 72 patients<br>- Arm B= 36 patients<br>- Metastatic renal cell carcinoma	CXCR4	- LY2510924 (LY) at 20mg/day as an SCI for 4 weeks.	- Sunitinib (SUN) at 50mg/day orally for 4 weeks, then 2 weeks during SUN were not administered.<br>- Arm A treatment range= 1-27 cycles.<br>- Arm B treatment range= 1-25 cycles	• Arm A: LY+SUN<br>- OR= 30.6 (19.92, 41.2)<br>- Median DR= 8.4 (95% C.I: 5.4-20.7)<br>- SD= 46%; PD= 18%; ORR= 31%<br>- Median PFS= 8.1 (95% C.I: 5.5-10.6)<br>- Median OS= 24.2 (95% C.I:13.9-NA)	• Arm B: SUN<br>- OR= 38.9 (22.96, 54.81)<br>- Median DR= 12.4 (95% C.I: 3.2-NA)<br>- SD= 33%; PD= 20%; ORR= 39%<br>- Median PFS= 12.3 (95% C.I: 2.7-20)<br>- Median OS= 24.9 (95% C.I: 11.6-NA)	The addition of LY to SUN did not improve the PFS or OS vs. SUN alone. CXCR4 remains an unproven therapeutic target for the treatment of renal cell carcinoma.
Biasci et al. (2020)[37]	- Multicenter, nonrandomized, open-label, Phase I, dose escalation trial<br>- Microsatellite stable colorectal cancer (n=15) and pancreatic ductal adenocarcinoma (n=9).<br>- Dose escalation phase n=17.<br>- Dose-expansion phase n=7.	CXCR4	- Plerixafor at continuous IV infusion rate at 20, 40, 80 y 120 µg/kg/h.<br>- MTD= 120 µg/kg/h.<br>- DLT= 80 µg/kg/h.	Not required	• CR or PR= 0%<br>• SD=13 (57%)<br>• PD= 10 (43%)	Not required	Most of the patients treated with Plerixaflor showed enhanced tumoral immune responses after only 7 days.

O`Hara et al. (2020)[47]	- Open-label, Phase Ia, dose escalation trial<br>- 3 patients for dose group<br>- Pancreatic and rectal carcinoma	CXCR4	- LY2510924 (LY), SCI at 20, 30 or 40 mg/day on 28-day cycle.<br>- DLT= not reported	- Durvalumab at 1500 mg, administered IV on day 1 of each 28-day cycle.<br>- Min. N° of cycles= 1<br>- Max. N° of cycles= 6	- SD= 4 (44%)<br>- PD= 5 (55%)<br>- PR or CR= 0 (0%)	Not required	The recommended phase 2 dose is 40mg SC once-daily LY2510924 in combination with durvalumab 1500mg IV and showed acceptable safety and tolerability in patients with advanced refractory tumors.
Bockorny et al. (2020)[80]	- Multicenter, open-label, two-cohort, Phase IIa trial<br>- Metastatic pancreatic adenocarcinoma	CXCR4	- Motixafortide at 1.25 mg kg−1 at SCI on days 1–5.<br>- During the combination phase, received 1.25 mg kg−1 at SCI 3 times a week.	• Pembrolizumab, IV:<br>- 200 mg once every 3 weeks.<br>• Chemotherapy, IV:<br>- every 2 weeks:<br>- LI: 70 mg per m^2 over 90 min<br>- LV: 400 mg per m^2 over 30 min<br>- FU: 2,400 mg per m^2 over 46 h	• Cohort 1:<br>- ORR=<br>- DCR= 34.5%<br>- Median OS= 3.3	• Cohort 2:<br>- ORR= 32%<br>- DCR= 77%<br>- Median DR= 7.8	These data suggest that Motixafortide and Pembrolizumab may expand the benefit of chemotherapy in pancreatic adenocarcinoma and merit confirmation in a subsequent randomized trial.
Bockorny et al. (2021)[81]	- Multicenter, single-arm, phase II trial.<br>- Metastatic pancreatic adenocarcinoma.<br>- n= 43 patients<br>- Only 38 patients received combination therapy and were evaluable for efficacy.	CXCR4	- Motixafortide at 1.25 mg/kg daily at SCI on days 1–5, during monotherapy period.<br>- Beginning on day 10, Motixafortide twice a week and at least 24 hours after chemotherapy dosing.	• Chemotherapy, IV:<br>- From day 8 and every 2 weeks:<br>- LI: 70 mg per m^2 over 90 min<br>- LV: 400 mg per m^2 over 30 min<br>- FU: 2,400 mg per m^2 over 46 h<br>• Pembrolizumab, IV:<br>- 200 mg once every 3 weeks.<br>- The combination therapy continued	• Intention to treat population (ITT):<br>- ORR= 13,2% (95% CI: 2.4-23.9)<br>- SD= 42.1% (95% CI: 26.4-57.8)<br>- DCR= 63,2% (95% CI: 47.8-78.5)<br>- Median DR= 5.7 (95% CI: 4.9-7.3)<br>- Median OS= 6.6 (95% CI: 4.5-8.7)<br>- Median PFS= 3.8 (95% CI: 1.6-5.1)	• Liver metastasis subgroup (n=30):<br>- ORR= 16.7% (95% CI: 3.3-30)<br>- DCR= 56.7% (95% CI: 38.9-74.4<br>- Median PFS= 1.9 (95% C.I: 1.5-5.7)<br>- Median OS= 5.9 (95% CI:4.4-9.6)<br>• Non-liver metastasis subgroup (n=8):<br>- ORR= 37.5 % (95% CI: 4.0-71)<br>- DCR= 87.5% (95% CI: 64.4-100)	Triple combination of Motixafortide, Pembrolizumab, and Chemotherapy was safe and well tolerated, and showed signs of efficacy in a population with poor prognosis and aggressive disease.

				for up to 35 cycles (approximately two years).		- Median PFS= 5.4 (95% C.I: 1.5-8.0)<br>- Median OS= 8.4 (95% CI:3.5-10.8)	
Choueiri et al. (2021)[82]	- Multicenter, open-label, Phase Ib trial<br>- N° of patients= 9<br>- Clear renal cell carcinoma	CXCR4	- Mavorixafor 400 mg, orally, daily for 28-day cycles.	- Nivolumab 240 mg as monotherapy (IV) every 2 weeks. The median duration of combined therapy was 3.7 months (1–15).	- SD= 7 (78%)<br>- PD= 1(11%)<br>- PR: 1 (11%)<br>- ORR: 11%<br>- Median DR:= 6.7 (3.7–14.7).	Not required	Combined therapy in patients with advanced clear renal cell carcinoma demonstrated potential antitumor activity and a manageable safety profile.
Halama et al. (2016)[106]	- Phase I<br>- Core cohort= 11 patients<br>- Extension cohort= 3 patients<br>- Metastatic colorectal carcinoma	CCR5	- Maraviroc 300 mg, orally, twice daily for 2 months.	Not required	• Core cohort:<br>- Median OS= 5.06 (95% C.I: 3.06-∞)<br>- Median PFS= 1.15 (95% C.I: 0.66-∞)<br>- DCR: 80%	• Extension cohort:<br>- Median PFS=1.55 (1.35, 1.55, 3.29)<br>- Median OS= 6.94 (6.91, 6.94, 16.08)<br>- DCR: 66%	CCR5 blockade showed clinical effects with regression of metastatic colorectal carcinoma without significant side effects.
Haag et al. (2022)[108]	- Phase I<br>- Total patients= 20<br>- Metastatic colorectal carcinoma	CCR5	- Maraviroc 300, orally, twice daily.	- Pembrolizumab 200 mg, IV, every 21 days in combination with Maraviroc for a maximum total of 8 cycles.	- PR= 1 (5,35%)<br>- PD= 18 (94,7%)<br>- ORR= 5.3%.<br>- PFS= 2.10 (95% CI: 1.68-2.30)<br>- Median OS= 9.83 (95% CI: 5.59-20)<br>- DCR: : >70%	Not required	Therapy with pembrolizumab and maraviroc was feasible and showed a beneficial toxicity pattern.<br>Clinical activity in mismatch repair proficient metastatic colorectal carcinoma patients was limited.
